# Bis(μ-2-fluoro­benzoato-1:2κ^2^ *O*:*O*′)(2-fluoro­benzoato-1κ^2^ *O*,*O*′)(2-fluoro­benzoato-2κ*O*)dinicotinamide-1κ*N* ^1^,2κ*N* ^1^-dizinc(II)–2-fluoro­benzoic acid (1/1)

**DOI:** 10.1107/S1600536809048089

**Published:** 2009-11-21

**Authors:** Tuncer Hökelek, Filiz Yılmaz, Barış Tercan, F. Elif Özbek, Hacali Necefoğlu

**Affiliations:** a Department of Physics, Hacettepe University, 06800 Beytepe, Ankara, Turkey; b Department of Chemistry, Faculty of Science, Anadolu University, 26470 Yenibağlar, Eskişehir, Turkey; c Department of Physics, Karabük University, 78050 Karabük, Turkey; d Department of Chemistry, Kafkas University, 63100 Kars, Turkey

## Abstract

The asymmetric unit of the title compound, [Zn_2_(C_7_H_4_FO_2_)_4_(C_6_H_6_N_2_O)_2_]·C_7_H_5_FO_2_, consists of a binuclear Zn^II^ complex bridged by two carboxyl groups of 2-fluoro­benzoate (FB) anions and a 2-fluoro­benzoic acid mol­ecule. The two bridging FB anions, one chelating FB anion and one nicotinamide (NA) ligand coordinate to one Zn cation with a distorted square-pyramidal geometry, while the two bridging FB anions, one monodentate FB anion and one NA ligand coordinate to the other Zn cation with a distorted tetra­hedral geometry. Within the binuclear mol­ecule, the pyridine rings are oriented at a dihedral angle of 19.41 (14)°. In the crystal structure, the uncoordinated 2-fluorobenzoic acid mol­ecules are linked by O—H⋯O hydrogen bonding, forming centrosymmetric supra­molecular dimers. Inter­molecular N—H⋯O hydrogen bonds link the complex mol­ecules into a three-dimensional network. The π–π contacts between nearly parallel pyridine and benzene rings [dihedral angles of 19.41 (14) and 12.72 (16)°, respectively, centroid–centroid distances = 3.701 (2) and 3.857 (3) Å] may further stabilize the crystal structure. The fluorine atoms in two FB ligands are disordered over two positions, with occupancy ratios of 0.70:0.30.

## Related literature

For general background to nicotinamide and the nicotinic acid derivative *N*,*N*-diethyl­nicotinamide, see: Bigoli *et al.* (1972 ▶); Krishnamachari (1974 ▶). For related structures, see: Hökelek & Necefoğlu (1996 ▶); Hökelek *et al.* (2009*a* ▶,*b* ▶,*c* ▶,*d* ▶); Greenaway *et al.* (1984 ▶).
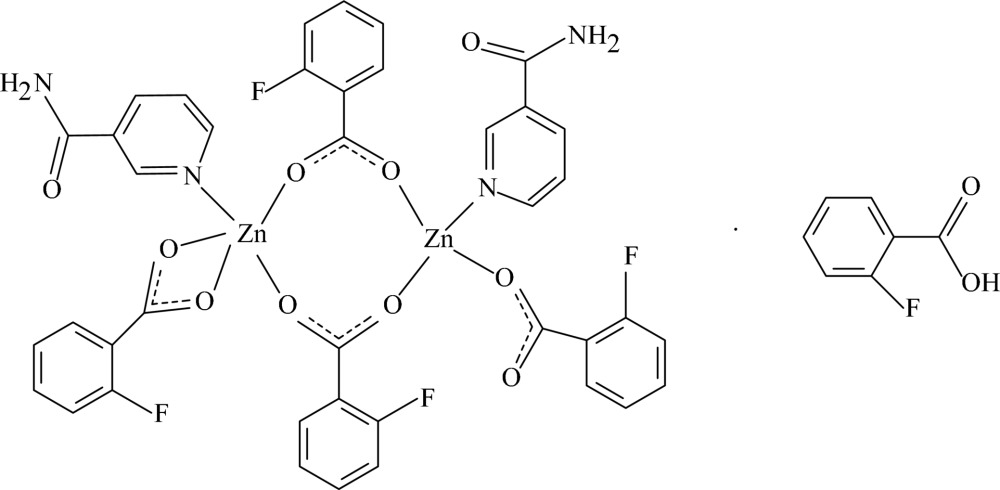


## Experimental

### 

#### Crystal data

[Zn_2_(C_7_H_4_FO_2_)_4_(C_6_H_6_N_2_O)_2_]·C_7_H_5_FO_2_
*M* *_r_* = 1071.55
Monoclinic, 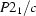

*a* = 12.5143 (2) Å
*b* = 16.7106 (3) Å
*c* = 20.6673 (4) Å
β = 92.929 (2)°
*V* = 4316.33 (13) Å^3^
*Z* = 4
Mo *K*α radiation
μ = 1.21 mm^−1^
*T* = 100 K
0.29 × 0.25 × 0.14 mm

#### Data collection

Bruker Kappa APEXII CCD area-detector diffractometer
Absorption correction: multi-scan (*SADABS*; Bruker, 2005 ▶) *T*_min_ = 0.710, *T*_max_ = 0.840
37814 measured reflections
10696 independent reflections
7459 reflections with *I* > 2σ(*I*)
*R*_int_ = 0.036

#### Refinement

*R*[*F* ^2^ > 2σ(*F* ^2^)] = 0.042
*wR*(*F* ^2^) = 0.097
*S* = 1.01
10696 reflections
659 parameters
10 restraints
H atoms treated by a mixture of independent and constrained refinement
Δρ_max_ = 1.39 e Å^−3^
Δρ_min_ = −0.59 e Å^−3^

### 

Data collection: *APEX2* (Bruker, 2007 ▶); cell refinement: *SAINT* (Bruker, 2007 ▶); data reduction: *SAINT*; program(s) used to solve structure: *SHELXS97* (Sheldrick, 2008 ▶); program(s) used to refine structure: *SHELXL97* (Sheldrick, 2008 ▶); molecular graphics: *ORTEP-3 for Windows* (Farrugia, 1997 ▶); software used to prepare material for publication: *WinGX* (Farrugia, 1999 ▶) and *PLATON* (Spek, 2009 ▶).

## Supplementary Material

Crystal structure: contains datablocks I, global. DOI: 10.1107/S1600536809048089/xu2668sup1.cif

Structure factors: contains datablocks I. DOI: 10.1107/S1600536809048089/xu2668Isup2.hkl

Additional supplementary materials:  crystallographic information; 3D view; checkCIF report

## Figures and Tables

**Table 1:** Selected bond lengths (Å)

Zn1—O1	2.296 (3)
Zn1—O2	2.006 (3)
Zn1—O5	1.958 (3)
Zn1—O7	2.005 (3)
Zn1—N1	2.068 (4)
Zn2—O3	1.995 (3)
Zn2—O6	1.975 (3)
Zn2—O8	1.940 (3)
Zn2—N3	2.021 (4)

**Table 2:** Hydrogen-bond geometry (Å, °)

*D*—H⋯*A*	*D*—H	H⋯*A*	*D*⋯*A*	*D*—H⋯*A*
N2—H2*A*⋯O1^i^	0.82 (5)	2.39 (5)	3.056 (6)	140 (5)
N2—H2*B*⋯O10^ii^	0.84 (6)	2.03 (5)	2.863 (6)	176.4 (5)
N4—H4*A*⋯O4^iii^	0.81 (5)	2.11 (5)	2.825 (5)	149 (5)
N4—H4*B*⋯O9^iv^	0.92 (7)	1.96 (7)	2.874 (6)	176 (7)
O12—H121⋯O11^v^	0.92 (8)	1.71 (8)	2.626 (5)	174 (6)

Symmetry codes: (i) 
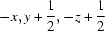
; (ii) 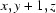; (iii) 
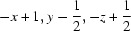
; (iv) 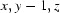; (v) 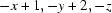.

**Fig. 1.**
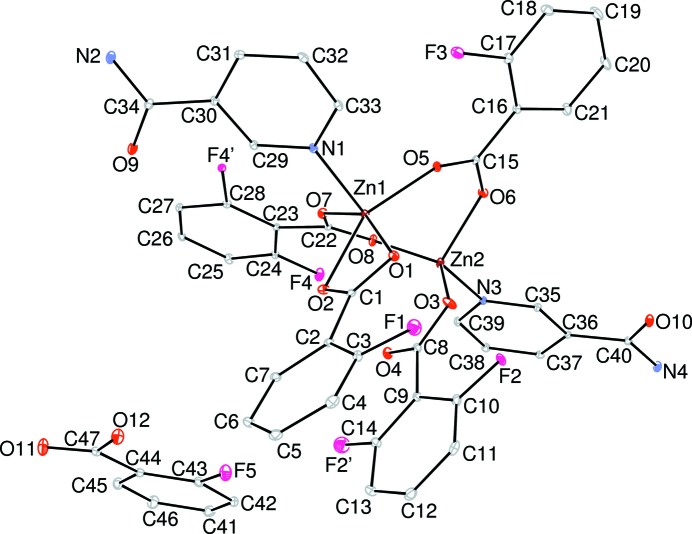
The molecular structure of the title molecule with the atom-numbering scheme. Displacement ellipsoids are drawn at the 20% probability level. Hydrogen atoms have been omitted for clarity.

## supplementary crystallographic information

### Comment

As a part of our ongoing investigation on transition metal complexes of nicotinamide (NA), one form of niacin (Krishnamachari, 1974), and/or the nicotinic acid derivative *N*,*N*-diethylnicotinamide (DENA), an important respiratory stimulant (Bigoli *et al.*, 1972), the title compound was synthesized and its crystal structure is reported herein.

The title compound is a binuclear compound, consisting of two nicotinamide (NA), four 2-fluorobenzoate (FB) ligands and one 2-fluorobenzoic acid molecule. The structures of some DENA and/or NA complexes of Zn^II^ ion, [Zn_2_(C_11_H_14_NO_2_)_4_(C_10_H_14_N_2_O)_2_] (Hökelek *et al.*, 2009*a*) and [Zn_2_(C_8_H_8_NO_2_)_4_(C_10_H_14_N_2_O)_2_].2H_2_O (Hökelek *et al.*, 2009*b*) have also been determined.

In the title dimeric complex, [Zn_2_(C_7_H_4_FO_2_)_4_(C_6_H_6_N_2_O)_2_].(C_7_H_5_FO_2_), two Zn^II^ atoms are surrounded by three FB groups and one NA ligand. The NA ligands are coordinated to Zn^II^ ions through pyridine N atoms only. Two FB groups act as bridging ligands, while the other two FB groups are coordinated to Zn^II^ ions bidentately and monodentately. The Zn1···Zn2 distance is 3.315 (3) Å. Atom Zn1 lies 0.2403 (5) Å below the best least-squares plane of the four O atoms (O1, O2, O5 and O7) [with a maximum deviation of 0.119 (3) Å for atom O2], while atom Zn2 lies 0.5735 (5) Å below the least-squares plane of the three O atoms (O3, O6 and O8). The four O atoms around the Zn1 atom form a distorted square-planar arrangement with an average Zn1—O bond length of 2.06625 (30) Å. The distorted square-pyramidal coordination is completed by the pyridine N1 atom of the NA ligand at a distance of 2.068 (4) Å (Table 1, Fig. 1). The three nearest O atoms arrangement [with an average Zn2—O bond length of 1.970 (3) Å] around Zn2 atom is completed by the pyridine N3 atom of the NA ligand at a distance of 2.021 (4) Å to form a tetrahedral coordinaton (Table 1, Fig. 1).

The N1—Zn1···Zn2 and N3—Zn2···Zn1 angles are 152.72 (3) and 171.99 (3) °, respectively. The dihedral angle between the best least-squares planes through Zn1, O5, C15, O6, Zn2 [with a maximum deviation of 0.115 (3) Å for atom O5] and Zn1, O7, C22, O8, Zn2 [with a maximum deviation of -0.089 (3) Å for atom O8] is 115.74 (5)°. The dihedral angles between the planar carboxylate groups (O1/O2/C1),(O3/O4/C8), (O5/O6/C15), (O7/O8/C22) and the adjacent benzene rings A (C2—C7), B (C9—C14), C (C16—C21), D (C23—C28) are 10.2 (5)°, 7.65 (17)°, 16.03 (30)° and 16.21 (35)°, respectively, while those between rings A, B, C and D are A/B = 12.72 (16), A/C = 71.97 (15), A/D = 71.15 (14), B/C = 78.14 (15), B/D = 83.60 (16) and C/D = 47.65 (13) °. The pyridine rings E (N1/C29—C33) and F (N3/C35—C39) are oriented at a dihedral angle of 19.41 (14)°. The O1—Zn1—O2 angle is 60.92 (12)°. The corresponding O—M—O (where *M* is a metal) angles are 58.3 (3)° in [Zn_2_(C_10_H_14_N_2_O)_2_(C_7_H_5_O_3_)_4_].2H_2_O (Hökelek & Necefoğlu, 1996), 60.03 (6)° in [Zn(C_9_H_10_NO_2_)_2_(C_6_H_6_N_2_O)(H_2_O)_2_] (Hökelek *et al.*, 2009*c*), 52.91 (4) and 53.96 (4) ° in [Cd(C_8_H_5_O_3_)_2_(C_6_H_6_N_2_O)_2_(H_2_O)].H_2_O (Hökelek *et al.*, 2009d) and 55.2 (1)° in [Cu(Asp)_2_(py)_2_] (where Asp is acetylsalicylate and py is pyridine) (Greenaway *et al.*, 1984).

In the crystal structure, N—H···O and O—H···O hydrogen bonds (Table 2) link the molecules into a three-dimensional network, in which they may be effective in the stabilization of the structure. The π–π contacts between the benzene and benzene rings, benzene and pyridine rings, pyridine and H (Zn1/O1/O2/C1) rings and pyridine and benzene rings, *Cg*1—*Cg*2, *Cg*4—*Cg*6^i^, *Cg*5—*Cg*8 and *Cg*6—*Cg*7^i^ [symmetry code: (i) 1 - *x*, *y* - 1/2, 1/2 - *z*, where *Cg*1, *Cg*2, *Cg*4, *Cg*5, *Cg*6, *Cg*7 and *Cg*8 are centroids of the rings A (C2—C7), B (C9—C14), D (C23—C28), E (N1/C29—C33), F (N3/C35—C39), G (C41—C46) and H (Zn1/O1/O2/C1), respectively] may further stabilize the structure, with centroid-centroid distances of 3.716 (3), 3.701 (2), 3.926 (2) and 3.857 (3) Å, respectively.

### Experimental

The title compound was prepared by the reaction of ZnSO_4_.H_2_O (0.89 g, 5 mmol) in H_2_O (20 ml) and NA (1.22 g, 10 mmol) in H_2_O (20 ml) with sodium 2-fluorobenzoate (1.62 g, 10 mmol) in H_2_O (50 ml). The mixture was filtered and set aside to crystallize at ambient temperature for two days, giving colorless single crystals.

### Refinement

Atoms H2A, H2B, H4A and H4B (for NH_2_) and H121 (for OH) were located in a difference Fourier map and refined isotropically. The remaining H atoms were positioned geometrically with C—H = 0.93 Å for aromatic H atoms and constrained to ride on their parent atoms, with *U*_iso_(H) = 1.2*U*_eq_(C). The F2 and H14 atoms attached at C10 and C14, and the F4 and H28 atoms attached at C24 and C28, respectively, are disordered over two orientations. During the refinement process, the disordered F2, F4, H14, H28 and F2', F4', H14', H28' atoms were refined with occupancies of 0.7 and 0.3, respectively.

### Crystal data

**Table:**

[Zn_2_(C_7_H_4_FO_2_)_4_(C_6_H_6_N_2_O)_2_]·C_7_H_5_FO_2_	*F*(000) = 2176
*M**_r_* = 1071.55	*D*_x_ = 1.649 Mg m^−^^3^
Monoclinic, *P*2_1_/*c*	Mo *K*α radiation, λ = 0.71073 Å
Hall symbol: -P 2ybc	Cell parameters from 9923 reflections
*a* = 12.5143 (2) Å	θ = 2.3–28.1°
*b* = 16.7106 (3) Å	µ = 1.21 mm^−^^1^
*c* = 20.6673 (4) Å	*T* = 100 K
β = 92.929 (2)°	Block, colorless
*V* = 4316.33 (13) Å^3^	0.29 × 0.25 × 0.14 mm
*Z* = 4	

### Data collection

**Table:**

Bruker Kappa APEXII CCD area-detector diffractometer	10696 independent reflections
Radiation source: fine-focus sealed tube	7459 reflections with *I* > 2σ(*I*)
graphite	*R*_int_ = 0.036
φ and ω scans	θ_max_ = 28.3°, θ_min_ = 1.6°
Absorption correction: multi-scan (*SADABS*; Bruker, 2005)	*h* = −16→13
*T*_min_ = 0.710, *T*_max_ = 0.840	*k* = −22→22
37814 measured reflections	*l* = −26→27

### Refinement

**Table:**

Refinement on *F*^2^	Primary atom site location: structure-invariant direct methods
Least-squares matrix: full	Secondary atom site location: difference Fourier map
*R*[*F*^2^ > 2σ(*F*^2^)] = 0.042	Hydrogen site location: inferred from neighbouring sites
*wR*(*F*^2^) = 0.097	H atoms treated by a mixture of independent and constrained refinement
*S* = 1.01	*w* = 1/[σ^2^(*F*_o_^2^) + (0.0383*P*)^2^ + 3.8099*P*] where *P* = (*F*_o_^2^ + 2*F*_c_^2^)/3
10696 reflections	(Δ/σ)_max_ = 0.001
659 parameters	Δρ_max_ = 1.39 e Å^−^^3^
10 restraints	Δρ_min_ = −0.59 e Å^−^^3^

### Special details

**Table:**

Geometry. All e.s.d.'s (except the e.s.d. in the dihedral angle between two l.s. planes) are estimated using the full covariance matrix. The cell e.s.d.'s are taken into account individually in the estimation of e.s.d.'s in distances, angles and torsion angles; correlations between e.s.d.'s in cell parameters are only used when they are defined by crystal symmetry. An approximate (isotropic) treatment of cell e.s.d.'s is used for estimating e.s.d.'s involving l.s. planes.
Refinement. Refinement of *F*^2^ against ALL reflections. The weighted *R*-factor *wR* and goodness of fit *S* are based on *F*^2^, conventional *R*-factors *R* are based on *F*, with *F* set to zero for negative *F*^2^. The threshold expression of *F*^2^ > σ(*F*^2^) is used only for calculating *R*-factors(gt) *etc*. and is not relevant to the choice of reflections for refinement. *R*-factors based on *F*^2^ are statistically about twice as large as those based on *F*, and *R*- factors based on ALL data will be even larger.

### Fractional atomic coordinates and isotropic or equivalent isotropic displacement parameters (Å^2^)

**Table:**

	*x*	*y*	*z*	*U*_iso_*/*U*_eq_	Occ. (<1)
Zn1	0.17153 (4)	0.67257 (3)	0.24483 (2)	0.01406 (14)	
Zn2	0.31257 (4)	0.51261 (3)	0.29078 (3)	0.01641 (14)	
F1	−0.0139 (2)	0.6414 (2)	0.02860 (15)	0.0383 (8)	
F2	0.1234 (3)	0.4736 (2)	0.09432 (18)	0.0236 (9)	0.70
F2'	0.4720 (5)	0.5834 (7)	0.0809 (5)	0.054 (3)*	0.30
F3	−0.0844 (2)	0.64099 (18)	0.36234 (15)	0.0362 (8)	
F4	0.6020 (3)	0.6154 (2)	0.3631 (2)	0.0291 (10)	0.70
F4'	0.3673 (4)	0.8400 (4)	0.3434 (4)	0.0138 (17)*	0.30
F5	0.4883 (2)	0.73739 (19)	0.00733 (15)	0.0377 (8)	
O1	0.0703 (3)	0.6540 (2)	0.15019 (15)	0.0240 (8)	
O2	0.2346 (2)	0.6999 (2)	0.16036 (14)	0.0199 (7)	
O3	0.2399 (2)	0.5160 (2)	0.20262 (16)	0.0270 (8)	
O4	0.4067 (2)	0.5492 (2)	0.19029 (16)	0.0242 (8)	
O5	0.0874 (2)	0.60130 (18)	0.29767 (14)	0.0163 (7)	
O6	0.1828 (2)	0.49874 (19)	0.34017 (15)	0.0199 (7)	
O7	0.3061 (2)	0.6981 (2)	0.29769 (14)	0.0182 (7)	
O8	0.4007 (2)	0.59306 (19)	0.33526 (16)	0.0211 (7)	
O9	0.2568 (3)	0.9875 (2)	0.26713 (19)	0.0319 (9)	
O10	0.2451 (2)	0.2018 (2)	0.27913 (16)	0.0228 (8)	
O11	0.6174 (3)	0.9659 (2)	0.0211 (2)	0.0380 (10)	
O12	0.4706 (3)	0.8936 (2)	0.00208 (19)	0.0331 (9)	
H121	0.443 (5)	0.943 (5)	−0.008 (3)	0.07 (2)*	
N1	0.0910 (3)	0.7776 (2)	0.26319 (17)	0.0148 (8)	
N2	0.1202 (4)	1.0617 (3)	0.3008 (2)	0.0235 (10)	
H2A	0.058 (4)	1.063 (3)	0.310 (2)	0.022 (14)*	
H2B	0.158 (4)	1.102 (3)	0.296 (3)	0.030 (16)*	
N3	0.4006 (3)	0.4123 (2)	0.30545 (17)	0.0142 (8)	
N4	0.3912 (4)	0.1241 (3)	0.2944 (2)	0.0209 (9)	
H4A	0.454 (4)	0.120 (3)	0.304 (2)	0.024 (15)*	
H4B	0.347 (5)	0.082 (4)	0.284 (3)	0.06 (2)*	
C1	0.1502 (4)	0.6839 (3)	0.1255 (2)	0.0172 (10)	
C2	0.1499 (3)	0.7054 (3)	0.0554 (2)	0.0163 (10)	
C3	0.0668 (4)	0.6878 (3)	0.0113 (2)	0.0203 (10)	
C4	0.0640 (4)	0.7140 (3)	−0.0521 (2)	0.0273 (12)	
H4	0.0060	0.7019	−0.0803	0.033*	
C5	0.1486 (4)	0.7584 (3)	−0.0729 (2)	0.0290 (12)	
H5	0.1472	0.7774	−0.1152	0.035*	
C6	0.2351 (4)	0.7745 (3)	−0.0310 (2)	0.0255 (12)	
H6	0.2931	0.8030	−0.0455	0.031*	
C7	0.2356 (4)	0.7483 (3)	0.0323 (2)	0.0196 (10)	
H7	0.2944	0.7595	0.0602	0.023*	
C8	0.3164 (3)	0.5334 (3)	0.1666 (2)	0.0179 (10)	
C9	0.2958 (3)	0.5326 (3)	0.0950 (2)	0.0169 (10)	
C10	0.2015 (4)	0.5058 (3)	0.0629 (2)	0.0230 (11)	
H14'	0.1473	0.4853	0.0872	0.028*	0.30
C11	0.1859 (4)	0.5086 (3)	−0.0046 (3)	0.0282 (12)	
H11	0.1222	0.4903	−0.0246	0.034*	
C12	0.2649 (4)	0.5383 (3)	−0.0407 (3)	0.0312 (13)	
H12	0.2543	0.5405	−0.0855	0.037*	
C13	0.3598 (4)	0.5651 (3)	−0.0124 (2)	0.0306 (13)	
H13	0.4132	0.5850	−0.0377	0.037*	
C14	0.3749 (4)	0.5620 (3)	0.0552 (2)	0.0234 (11)	
H14	0.4394	0.5799	0.0744	0.028*	0.70
C15	0.0994 (3)	0.5414 (3)	0.3341 (2)	0.0145 (9)	
C16	0.0079 (3)	0.5168 (3)	0.3735 (2)	0.0164 (10)	
C17	−0.0787 (4)	0.5654 (3)	0.3857 (2)	0.0218 (11)	
C18	−0.1606 (4)	0.5413 (3)	0.4231 (2)	0.0286 (12)	
H18	−0.2176	0.5754	0.4301	0.034*	
C19	−0.1571 (4)	0.4659 (4)	0.4501 (3)	0.0362 (14)	
H19	−0.2119	0.4489	0.4756	0.043*	
C20	−0.0727 (4)	0.4155 (3)	0.4393 (3)	0.0367 (14)	
H20	−0.0700	0.3647	0.4576	0.044*	
C21	0.0082 (4)	0.4410 (3)	0.4010 (2)	0.0258 (12)	
H21	0.0644	0.4064	0.3934	0.031*	
C22	0.3881 (3)	0.6668 (3)	0.3252 (2)	0.0158 (10)	
C23	0.4772 (3)	0.7209 (3)	0.3453 (2)	0.0141 (9)	
C24	0.5816 (3)	0.6927 (3)	0.3596 (2)	0.0174 (10)	
H28'	0.5940	0.6378	0.3605	0.021*	0.30
C25	0.6675 (4)	0.7452 (3)	0.3726 (2)	0.0216 (11)	
H25	0.7362	0.7253	0.3811	0.026*	
C26	0.6492 (4)	0.8258 (3)	0.3728 (2)	0.0216 (11)	
H26	0.7064	0.8605	0.3812	0.026*	
C27	0.5482 (4)	0.8570 (3)	0.3608 (2)	0.0214 (11)	
H27	0.5364	0.9119	0.3621	0.026*	
C28	0.4637 (3)	0.8040 (3)	0.3467 (2)	0.0186 (10)	
H28	0.3957	0.8248	0.3378	0.022*	0.70
C29	0.1429 (3)	0.8473 (3)	0.2675 (2)	0.0146 (9)	
H29	0.2162	0.8472	0.2620	0.018*	
C30	0.0941 (3)	0.9198 (3)	0.2797 (2)	0.0150 (10)	
C31	−0.0155 (3)	0.9193 (3)	0.2884 (2)	0.0168 (10)	
H31	−0.0515	0.9665	0.2972	0.020*	
C32	−0.0698 (3)	0.8475 (3)	0.2837 (2)	0.0181 (10)	
H32	−0.1430	0.8459	0.2895	0.022*	
C33	−0.0157 (3)	0.7782 (3)	0.2707 (2)	0.0168 (10)	
H33	−0.0536	0.7304	0.2668	0.020*	
C34	0.1630 (3)	0.9929 (3)	0.2819 (2)	0.0187 (10)	
C35	0.3537 (3)	0.3409 (3)	0.2987 (2)	0.0142 (9)	
H35	0.2799	0.3393	0.2906	0.017*	
C36	0.4082 (3)	0.2690 (3)	0.3032 (2)	0.0133 (9)	
C37	0.5183 (3)	0.2716 (3)	0.3166 (2)	0.0170 (10)	
H37	0.5579	0.2246	0.3204	0.020*	
C38	0.5676 (3)	0.3453 (3)	0.3241 (2)	0.0184 (10)	
H38	0.6409	0.3483	0.3336	0.022*	
C39	0.5075 (3)	0.4143 (3)	0.3175 (2)	0.0174 (10)	
H39	0.5418	0.4636	0.3214	0.021*	
C40	0.3426 (3)	0.1945 (3)	0.2915 (2)	0.0169 (10)	
C41	0.7570 (4)	0.6917 (3)	0.0614 (3)	0.0303 (12)	
H41	0.7990	0.6469	0.0709	0.036*	
C42	0.6512 (4)	0.6824 (3)	0.0410 (2)	0.0269 (12)	
H42	0.6215	0.6316	0.0367	0.032*	
C43	0.5899 (4)	0.7494 (3)	0.0272 (2)	0.0225 (11)	
C44	0.6319 (4)	0.8263 (3)	0.0324 (2)	0.0195 (10)	
C45	0.7393 (4)	0.8332 (3)	0.0534 (2)	0.0268 (12)	
H45	0.7699	0.8838	0.0578	0.032*	
C46	0.8006 (4)	0.7669 (3)	0.0677 (3)	0.0329 (13)	
H46	0.8719	0.7729	0.0817	0.039*	
C47	0.5720 (4)	0.9010 (3)	0.0178 (2)	0.0214 (11)	

### Atomic displacement parameters (Å^2^)

**Table:**

	*U*^11^	*U*^22^	*U*^33^	*U*^12^	*U*^13^	*U*^23^
Zn1	0.0128 (2)	0.0158 (3)	0.0136 (3)	−0.0009 (2)	0.00059 (19)	0.0017 (2)
Zn2	0.0136 (2)	0.0104 (3)	0.0248 (3)	0.0005 (2)	−0.0018 (2)	0.0001 (2)
F1	0.0310 (16)	0.042 (2)	0.0416 (19)	−0.0084 (15)	−0.0021 (14)	−0.0026 (16)
F2	0.0158 (18)	0.029 (2)	0.026 (2)	−0.0116 (17)	0.0042 (16)	0.0000 (18)
F3	0.0401 (17)	0.0233 (17)	0.0473 (19)	0.0134 (14)	0.0224 (15)	0.0138 (15)
F4	0.025 (2)	0.018 (2)	0.044 (3)	0.0016 (18)	−0.0054 (18)	0.003 (2)
F5	0.0316 (16)	0.0317 (19)	0.049 (2)	−0.0060 (14)	−0.0037 (14)	0.0023 (16)
O1	0.0318 (18)	0.021 (2)	0.0194 (17)	−0.0047 (16)	0.0082 (14)	0.0002 (15)
O2	0.0195 (15)	0.0255 (19)	0.0146 (16)	0.0056 (15)	−0.0014 (13)	0.0000 (15)
O3	0.0132 (15)	0.044 (2)	0.0240 (18)	0.0029 (16)	0.0032 (13)	0.0131 (17)
O4	0.0199 (16)	0.024 (2)	0.0287 (19)	−0.0058 (15)	−0.0002 (14)	−0.0004 (16)
O5	0.0183 (15)	0.0118 (17)	0.0189 (16)	−0.0005 (13)	0.0019 (13)	0.0034 (14)
O6	0.0169 (15)	0.0185 (19)	0.0246 (18)	0.0032 (14)	0.0033 (13)	0.0032 (14)
O7	0.0148 (15)	0.0205 (18)	0.0189 (17)	−0.0008 (14)	−0.0040 (13)	−0.0011 (14)
O8	0.0203 (16)	0.0134 (18)	0.0292 (19)	−0.0025 (14)	−0.0035 (14)	−0.0016 (15)
O9	0.0190 (16)	0.0164 (19)	0.061 (3)	−0.0025 (15)	0.0111 (17)	−0.0072 (18)
O10	0.0151 (15)	0.0142 (18)	0.039 (2)	0.0004 (14)	−0.0032 (14)	0.0005 (16)
O11	0.0239 (18)	0.022 (2)	0.068 (3)	−0.0010 (17)	0.0012 (18)	0.005 (2)
O12	0.0241 (18)	0.025 (2)	0.050 (3)	0.0028 (17)	−0.0048 (17)	0.0043 (19)
N1	0.0137 (17)	0.017 (2)	0.0136 (19)	−0.0028 (16)	−0.0004 (14)	0.0001 (16)
N2	0.015 (2)	0.015 (2)	0.040 (3)	0.001 (2)	−0.0005 (19)	0.000 (2)
N3	0.0141 (17)	0.013 (2)	0.0158 (19)	0.0004 (16)	0.0007 (14)	−0.0001 (16)
N4	0.017 (2)	0.013 (2)	0.032 (2)	0.0016 (19)	−0.0005 (18)	−0.0005 (19)
C1	0.024 (2)	0.012 (2)	0.016 (2)	0.005 (2)	0.0054 (19)	−0.001 (2)
C2	0.018 (2)	0.015 (2)	0.016 (2)	0.003 (2)	0.0017 (18)	−0.002 (2)
C3	0.022 (2)	0.018 (3)	0.021 (2)	−0.003 (2)	0.0013 (19)	−0.001 (2)
C4	0.035 (3)	0.027 (3)	0.019 (3)	0.003 (2)	−0.010 (2)	−0.004 (2)
C5	0.048 (3)	0.026 (3)	0.013 (2)	0.006 (3)	0.004 (2)	0.001 (2)
C6	0.029 (3)	0.026 (3)	0.022 (3)	0.000 (2)	0.010 (2)	0.002 (2)
C7	0.021 (2)	0.019 (3)	0.019 (2)	0.003 (2)	0.0038 (19)	−0.001 (2)
C8	0.018 (2)	0.011 (2)	0.024 (3)	0.0041 (19)	0.0036 (19)	0.003 (2)
C9	0.017 (2)	0.011 (2)	0.023 (2)	0.0040 (19)	0.0033 (18)	0.003 (2)
C10	0.023 (2)	0.013 (3)	0.033 (3)	0.001 (2)	0.003 (2)	0.001 (2)
C11	0.031 (3)	0.016 (3)	0.036 (3)	−0.001 (2)	−0.011 (2)	−0.006 (2)
C12	0.047 (3)	0.024 (3)	0.022 (3)	0.000 (3)	−0.001 (2)	−0.003 (2)
C13	0.038 (3)	0.031 (3)	0.024 (3)	−0.004 (3)	0.012 (2)	0.002 (2)
C14	0.021 (2)	0.023 (3)	0.027 (3)	−0.001 (2)	0.006 (2)	0.000 (2)
C15	0.016 (2)	0.013 (2)	0.015 (2)	−0.0024 (19)	−0.0026 (17)	−0.003 (2)
C16	0.016 (2)	0.015 (2)	0.017 (2)	0.000 (2)	−0.0012 (17)	0.001 (2)
C17	0.025 (2)	0.017 (3)	0.024 (3)	0.003 (2)	0.003 (2)	0.005 (2)
C18	0.023 (2)	0.030 (3)	0.033 (3)	0.009 (2)	0.010 (2)	0.009 (3)
C19	0.025 (3)	0.035 (3)	0.049 (4)	0.001 (3)	0.015 (2)	0.018 (3)
C20	0.030 (3)	0.024 (3)	0.058 (4)	0.000 (2)	0.014 (3)	0.021 (3)
C21	0.019 (2)	0.017 (3)	0.041 (3)	0.002 (2)	0.003 (2)	0.006 (2)
C22	0.018 (2)	0.017 (3)	0.012 (2)	−0.002 (2)	0.0047 (17)	−0.003 (2)
C23	0.019 (2)	0.013 (2)	0.010 (2)	−0.0023 (19)	0.0013 (17)	−0.0009 (19)
C24	0.019 (2)	0.015 (3)	0.018 (2)	−0.002 (2)	−0.0016 (18)	0.002 (2)
C25	0.018 (2)	0.025 (3)	0.022 (3)	−0.002 (2)	−0.0028 (19)	0.003 (2)
C26	0.021 (2)	0.021 (3)	0.023 (2)	−0.011 (2)	0.0035 (19)	−0.003 (2)
C27	0.028 (2)	0.014 (3)	0.023 (3)	−0.004 (2)	0.009 (2)	−0.003 (2)
C28	0.019 (2)	0.019 (3)	0.018 (2)	0.000 (2)	0.0037 (18)	−0.001 (2)
C29	0.0118 (19)	0.016 (3)	0.015 (2)	−0.0012 (19)	−0.0009 (17)	0.0019 (19)
C30	0.014 (2)	0.015 (2)	0.016 (2)	0.0012 (19)	−0.0018 (17)	0.0013 (19)
C31	0.017 (2)	0.019 (3)	0.015 (2)	0.004 (2)	0.0007 (18)	0.002 (2)
C32	0.0097 (19)	0.027 (3)	0.018 (2)	0.001 (2)	0.0000 (17)	0.005 (2)
C33	0.015 (2)	0.019 (3)	0.016 (2)	−0.004 (2)	−0.0022 (17)	0.004 (2)
C34	0.018 (2)	0.015 (3)	0.022 (2)	0.003 (2)	−0.0029 (19)	0.000 (2)
C35	0.0138 (19)	0.014 (2)	0.015 (2)	0.0004 (19)	−0.0006 (17)	0.0002 (19)
C36	0.016 (2)	0.012 (2)	0.012 (2)	0.0012 (19)	0.0012 (17)	−0.0001 (18)
C37	0.017 (2)	0.017 (3)	0.016 (2)	0.005 (2)	0.0019 (18)	0.002 (2)
C38	0.013 (2)	0.021 (3)	0.021 (2)	0.000 (2)	−0.0008 (18)	0.001 (2)
C39	0.017 (2)	0.016 (3)	0.019 (2)	−0.004 (2)	−0.0001 (18)	−0.001 (2)
C40	0.018 (2)	0.015 (3)	0.018 (2)	0.002 (2)	0.0024 (18)	0.000 (2)
C41	0.033 (3)	0.027 (3)	0.032 (3)	0.007 (2)	0.006 (2)	0.001 (2)
C42	0.040 (3)	0.018 (3)	0.024 (3)	−0.004 (2)	0.010 (2)	−0.001 (2)
C43	0.022 (2)	0.028 (3)	0.018 (2)	−0.004 (2)	0.0018 (19)	−0.001 (2)
C44	0.021 (2)	0.022 (3)	0.016 (2)	0.001 (2)	0.0037 (18)	−0.001 (2)
C45	0.025 (2)	0.023 (3)	0.033 (3)	0.000 (2)	0.002 (2)	0.000 (2)
C46	0.027 (3)	0.029 (3)	0.042 (3)	0.003 (3)	0.000 (2)	−0.001 (3)
C47	0.023 (2)	0.023 (3)	0.018 (2)	0.000 (2)	0.0057 (19)	0.000 (2)

### Geometric parameters (Å, °)

**Table:**

Zn1—O1	2.296 (3)	C13—H13	0.9300
Zn1—O2	2.006 (3)	C14—C13	1.402 (7)
Zn1—O5	1.958 (3)	C14—H14	0.9300
Zn1—O7	2.005 (3)	C16—C15	1.496 (6)
Zn1—N1	2.068 (4)	C16—C17	1.389 (6)
Zn2—O3	1.995 (3)	C16—C21	1.387 (7)
Zn2—O6	1.975 (3)	C18—C17	1.375 (6)
Zn2—O8	1.940 (3)	C18—C19	1.378 (7)
Zn2—N3	2.021 (4)	C18—H18	0.9300
F1—C3	1.338 (5)	C19—C20	1.378 (7)
F3—C17	1.353 (6)	C19—H19	0.9300
F5—C43	1.331 (5)	C20—H20	0.9300
O1—C1	1.250 (5)	C21—C20	1.384 (7)
O2—C1	1.276 (5)	C21—H21	0.9300
O3—C8	1.276 (5)	C22—C23	1.479 (6)
O4—C8	1.237 (5)	C24—C23	1.406 (6)
O5—C15	1.256 (5)	C24—H28'	0.9300
O6—C15	1.266 (5)	C25—C24	1.403 (6)
O7—C22	1.261 (5)	C25—H25	0.9300
O8—C22	1.259 (5)	C26—C25	1.367 (7)
O9—C34	1.231 (5)	C26—C27	1.378 (7)
O10—C40	1.240 (5)	C26—H26	0.9300
O11—C47	1.225 (6)	C27—C28	1.398 (6)
O12—C47	1.301 (6)	C27—H27	0.9300
O12—H121	0.92 (8)	C28—C23	1.398 (6)
N1—C29	1.335 (6)	C28—H28	0.9300
N1—C33	1.353 (5)	C29—C30	1.387 (6)
N2—C34	1.335 (6)	C29—H29	0.9300
N2—H2A	0.82 (5)	C31—C30	1.392 (6)
N2—H2B	0.84 (6)	C31—C32	1.380 (7)
N3—C35	1.335 (6)	C31—H31	0.9300
N3—C39	1.349 (5)	C32—H32	0.9300
N4—C40	1.324 (6)	C33—C32	1.374 (6)
N4—H4A	0.81 (5)	C33—H33	0.9300
N4—H4B	0.92 (7)	C34—C30	1.494 (6)
C2—C1	1.494 (6)	C35—H35	0.9300
C2—C3	1.379 (6)	C36—C35	1.382 (6)
C2—C7	1.394 (6)	C36—C37	1.391 (6)
C3—C4	1.379 (7)	C36—C40	1.504 (6)
C4—H4	0.9300	C37—C38	1.382 (6)
C5—C4	1.379 (7)	C37—H37	0.9300
C5—H5	0.9300	C38—H38	0.9300
C6—C5	1.378 (7)	C39—C38	1.380 (6)
C6—H6	0.9300	C39—H39	0.9300
C7—C6	1.380 (6)	C41—C46	1.374 (8)
C7—H7	0.9300	C41—H41	0.9300
C8—C9	1.490 (6)	C42—C41	1.377 (7)
C9—C14	1.407 (6)	C42—H42	0.9300
C10—C9	1.398 (6)	C43—C42	1.379 (7)
C10—C11	1.398 (7)	C44—C43	1.391 (7)
C10—H14'	0.9300	C44—C47	1.479 (7)
C11—H11	0.9300	C45—C44	1.395 (6)
C12—C11	1.363 (7)	C45—C46	1.371 (7)
C12—H12	0.9300	C45—H45	0.9300
C13—C12	1.372 (7)	C46—H46	0.9300
			
O2—Zn1—O1	60.92 (12)	C19—C18—H18	120.4
O2—Zn1—N1	100.57 (14)	C18—C19—C20	120.1 (5)
O5—Zn1—O1	95.98 (12)	C18—C19—H19	120.0
O5—Zn1—O2	150.56 (13)	C20—C19—H19	120.0
O5—Zn1—O7	106.60 (12)	C19—C20—C21	119.6 (5)
O5—Zn1—N1	97.64 (13)	C19—C20—H20	120.2
O7—Zn1—O1	154.68 (12)	C21—C20—H20	120.2
O7—Zn1—O2	93.94 (12)	C16—C21—H21	119.0
O7—Zn1—N1	97.05 (13)	C20—C21—C16	121.9 (5)
N1—Zn1—O1	91.04 (13)	C20—C21—H21	119.0
O3—Zn2—N3	112.33 (15)	O7—C22—C23	117.3 (4)
O6—Zn2—O3	97.28 (12)	O8—C22—O7	124.9 (4)
O6—Zn2—N3	106.36 (14)	O8—C22—C23	117.7 (4)
O8—Zn2—O3	129.40 (14)	C24—C23—C22	122.2 (4)
O8—Zn2—O6	107.39 (13)	C28—C23—C22	121.5 (4)
O8—Zn2—N3	102.19 (14)	C28—C23—C24	116.2 (4)
C1—O1—Zn1	82.8 (3)	C23—C24—H28'	119.2
C1—O2—Zn1	95.3 (3)	C25—C24—C23	121.6 (4)
C8—O3—Zn2	102.6 (3)	C25—C24—H28'	119.2
C15—O5—Zn1	140.1 (3)	C24—C25—H25	120.3
C15—O6—Zn2	125.3 (3)	C26—C25—C24	119.4 (4)
C22—O7—Zn1	143.0 (3)	C26—C25—H25	120.3
C22—O8—Zn2	122.4 (3)	C25—C26—C27	121.6 (4)
C47—O12—H121	109 (4)	C25—C26—H26	119.2
C29—N1—Zn1	120.9 (3)	C27—C26—H26	119.2
C29—N1—C33	117.7 (4)	C26—C27—C28	118.4 (4)
C33—N1—Zn1	121.4 (3)	C26—C27—H27	120.8
C34—N2—H2A	120 (4)	C28—C27—H27	120.8
C34—N2—H2B	115 (4)	C23—C28—C27	122.8 (4)
H2A—N2—H2B	124 (5)	C23—C28—H28	118.6
C35—N3—Zn2	119.5 (3)	C27—C28—H28	118.6
C35—N3—C39	118.0 (4)	N1—C29—C30	123.9 (4)
C39—N3—Zn2	122.4 (3)	N1—C29—H29	118.0
C40—N4—H4A	122 (4)	C30—C29—H29	118.0
C40—N4—H4B	114 (4)	C29—C30—C31	117.7 (4)
H4A—N4—H4B	124 (5)	C29—C30—C34	117.4 (4)
O1—C1—O2	120.7 (4)	C31—C30—C34	124.9 (4)
O1—C1—C2	122.0 (4)	C30—C31—H31	120.6
O2—C1—C2	117.3 (4)	C32—C31—C30	118.7 (4)
C3—C2—C1	123.7 (4)	C32—C31—H31	120.6
C3—C2—C7	116.8 (4)	C31—C32—H32	120.0
C7—C2—C1	119.5 (4)	C33—C32—C31	120.1 (4)
F1—C3—C2	120.1 (4)	C33—C32—H32	120.0
F1—C3—C4	117.1 (4)	N1—C33—C32	121.9 (4)
C4—C3—C2	122.8 (4)	N1—C33—H33	119.1
C3—C4—H4	120.5	C32—C33—H33	119.1
C5—C4—C3	118.9 (5)	O9—C34—N2	122.5 (4)
C5—C4—H4	120.5	O9—C34—C30	119.3 (4)
C4—C5—H5	120.0	N2—C34—C30	118.3 (4)
C6—C5—C4	120.1 (5)	N3—C35—C36	123.8 (4)
C6—C5—H5	120.0	N3—C35—H35	118.1
C5—C6—C7	119.9 (5)	C36—C35—H35	118.1
C5—C6—H6	120.1	C35—C36—C37	117.9 (4)
C7—C6—H6	120.1	C35—C36—C40	116.4 (4)
C2—C7—H7	119.3	C37—C36—C40	125.7 (4)
C6—C7—C2	121.4 (4)	C36—C37—H37	120.6
C6—C7—H7	119.3	C38—C37—C36	118.8 (4)
O3—C8—C9	118.7 (4)	C38—C37—H37	120.6
O4—C8—O3	121.1 (4)	C37—C38—H38	120.1
O4—C8—C9	120.2 (4)	C39—C38—C37	119.8 (4)
C10—C9—C8	125.0 (4)	C39—C38—H38	120.1
C14—C9—C10	115.9 (4)	N3—C39—C38	121.8 (4)
C14—C9—C8	119.1 (4)	N3—C39—H39	119.1
C9—C10—H14'	118.9	C38—C39—H39	119.1
C11—C10—C9	122.3 (4)	O10—C40—N4	122.8 (4)
C11—C10—H14'	118.9	O10—C40—C36	118.3 (4)
C10—C11—H11	120.3	N4—C40—C36	118.9 (4)
C12—C11—C10	119.4 (5)	C42—C41—H41	119.9
C12—C11—H11	120.3	C46—C41—C42	120.2 (5)
C11—C12—C13	121.5 (5)	C46—C41—H41	119.9
C11—C12—H12	119.3	C41—C42—C43	119.2 (5)
C13—C12—H12	119.3	C41—C42—H42	120.4
C12—C13—C14	118.9 (5)	C43—C42—H42	120.4
C12—C13—H13	120.6	F5—C43—C42	117.0 (5)
C14—C13—H13	120.6	F5—C43—C44	121.0 (4)
C9—C14—C13	122.2 (4)	C42—C43—C44	122.0 (4)
C9—C14—H14	118.9	C43—C44—C45	117.1 (5)
C13—C14—H14	118.9	C43—C44—C47	125.3 (4)
O5—C15—O6	125.5 (4)	C45—C44—C47	117.6 (4)
O5—C15—C16	118.4 (4)	C44—C45—H45	119.4
O6—C15—C16	116.1 (4)	C46—C45—C44	121.3 (5)
C17—C16—C15	124.3 (4)	C46—C45—H45	119.4
C21—C16—C15	119.2 (4)	C41—C46—H46	119.9
C21—C16—C17	116.4 (4)	C45—C46—C41	120.3 (5)
F3—C17—C16	120.6 (4)	C45—C46—H46	119.9
F3—C17—C18	116.6 (4)	O11—C47—O12	122.8 (5)
C18—C17—C16	122.8 (5)	O11—C47—C44	120.5 (4)
C17—C18—C19	119.1 (5)	O12—C47—C44	116.7 (5)
C17—C18—H18	120.4		
			
O2—Zn1—O1—C1	−3.1 (3)	C2—C7—C6—C5	0.0 (7)
O5—Zn1—O1—C1	−163.8 (3)	O3—C8—C9—C10	−7.4 (7)
O7—Zn1—O1—C1	−10.6 (4)	O3—C8—C9—C14	171.5 (4)
N1—Zn1—O1—C1	98.4 (3)	O4—C8—C9—C10	171.3 (4)
O1—Zn1—O2—C1	3.0 (2)	O4—C8—C9—C14	−9.7 (7)
O5—Zn1—O2—C1	45.1 (4)	C10—C9—C14—C13	0.8 (7)
O7—Zn1—O2—C1	179.9 (3)	C8—C9—C14—C13	−178.3 (5)
N1—Zn1—O2—C1	−82.2 (3)	C11—C10—C9—C14	−0.7 (7)
O1—Zn1—O5—C15	125.1 (5)	C11—C10—C9—C8	178.3 (5)
O2—Zn1—O5—C15	89.0 (5)	C9—C10—C11—C12	0.1 (8)
O7—Zn1—O5—C15	−43.3 (5)	C13—C12—C11—C10	0.4 (8)
N1—Zn1—O5—C15	−143.1 (5)	C14—C13—C12—C11	−0.4 (8)
O1—Zn1—O7—C22	−91.5 (5)	C9—C14—C13—C12	−0.3 (8)
O2—Zn1—O7—C22	−98.0 (5)	C17—C16—C15—O5	−17.1 (7)
O5—Zn1—O7—C22	60.6 (5)	C17—C16—C15—O6	163.6 (4)
N1—Zn1—O7—C22	160.8 (5)	C21—C16—C15—O5	163.9 (4)
O1—Zn1—N1—C29	−118.6 (3)	C21—C16—C15—O6	−15.4 (6)
O1—Zn1—N1—C33	60.8 (3)	C15—C16—C17—F3	−0.8 (7)
O2—Zn1—N1—C29	−58.0 (3)	C15—C16—C17—C18	−178.8 (5)
O2—Zn1—N1—C33	121.4 (3)	C21—C16—C17—F3	178.2 (4)
O5—Zn1—N1—C29	145.2 (3)	C21—C16—C17—C18	0.2 (7)
O5—Zn1—N1—C33	−35.4 (3)	C17—C16—C21—C20	−0.8 (7)
O7—Zn1—N1—C29	37.3 (3)	C15—C16—C21—C20	178.3 (5)
O7—Zn1—N1—C33	−143.2 (3)	C19—C18—C17—F3	−177.8 (5)
O8—Zn2—O3—C8	53.8 (3)	C19—C18—C17—C16	0.3 (8)
O6—Zn2—O3—C8	173.4 (3)	C17—C18—C19—C20	−0.3 (9)
N3—Zn2—O3—C8	−75.5 (3)	C18—C19—C20—C21	−0.3 (9)
O3—Zn2—O6—C15	−50.5 (4)	C16—C21—C20—C19	0.9 (9)
O8—Zn2—O6—C15	84.8 (4)	O7—C22—C23—C24	−162.1 (4)
N3—Zn2—O6—C15	−166.4 (3)	O7—C22—C23—C28	13.1 (6)
O3—Zn2—O8—C22	29.6 (4)	O8—C22—C23—C24	16.2 (6)
O6—Zn2—O8—C22	−85.8 (3)	O8—C22—C23—C28	−168.5 (4)
N3—Zn2—O8—C22	162.5 (3)	C25—C24—C23—C28	−1.8 (6)
O3—Zn2—N3—C35	−57.3 (3)	C25—C24—C23—C22	173.8 (4)
O3—Zn2—N3—C39	117.9 (3)	C26—C25—C24—C23	1.4 (7)
O6—Zn2—N3—C35	48.0 (3)	C27—C26—C25—C24	0.3 (7)
O6—Zn2—N3—C39	−136.8 (3)	C25—C26—C27—C28	−1.6 (7)
O8—Zn2—N3—C35	160.4 (3)	C26—C27—C28—C23	1.1 (7)
O8—Zn2—N3—C39	−24.3 (4)	C27—C28—C23—C24	0.5 (6)
Zn1—O1—C1—O2	5.0 (4)	C27—C28—C23—C22	−175.1 (4)
Zn1—O1—C1—C2	−172.5 (4)	N1—C29—C30—C31	0.5 (7)
Zn1—O2—C1—O1	−5.7 (5)	N1—C29—C30—C34	−178.9 (4)
Zn1—O2—C1—C2	171.9 (3)	C32—C31—C30—C29	−0.8 (6)
Zn2—O3—C8—O4	−5.4 (5)	C32—C31—C30—C34	178.7 (4)
Zn2—O3—C8—C9	173.4 (3)	C30—C31—C32—C33	−0.1 (6)
Zn1—O5—C15—O6	−11.4 (8)	N1—C33—C32—C31	1.3 (7)
Zn1—O5—C15—C16	169.4 (3)	N2—C34—C30—C29	−172.6 (4)
Zn2—O6—C15—O5	−3.1 (6)	N2—C34—C30—C31	8.0 (7)
Zn2—O6—C15—C16	176.2 (3)	O9—C34—C30—C29	6.7 (7)
Zn1—O7—C22—O8	−12.9 (7)	O9—C34—C30—C31	−172.8 (4)
Zn1—O7—C22—C23	165.3 (3)	C37—C36—C35—N3	1.1 (6)
Zn2—O8—C22—O7	16.4 (6)	C40—C36—C35—N3	−177.4 (4)
Zn2—O8—C22—C23	−161.8 (3)	C35—C36—C37—C38	−0.6 (6)
Zn1—N1—C29—C30	−180.0 (3)	C40—C36—C37—C38	177.9 (4)
C33—N1—C29—C30	0.6 (6)	C35—C36—C40—O10	−0.5 (6)
Zn1—N1—C33—C32	179.1 (3)	C37—C36—C40—O10	−179.0 (4)
C29—N1—C33—C32	−1.5 (6)	C35—C36—C40—N4	179.2 (4)
Zn2—N3—C35—C36	175.3 (3)	C37—C36—C40—N4	0.7 (7)
C39—N3—C35—C36	−0.2 (6)	C36—C37—C38—C39	−0.8 (6)
Zn2—N3—C39—C38	−176.6 (3)	N3—C39—C38—C37	1.8 (7)
C35—N3—C39—C38	−1.3 (6)	C42—C41—C46—C45	−0.4 (8)
C3—C2—C1—O1	−6.1 (7)	C43—C42—C41—C46	−0.1 (8)
C3—C2—C1—O2	176.3 (4)	F5—C43—C42—C41	179.6 (4)
C7—C2—C1—O1	171.4 (4)	C44—C43—C42—C41	0.8 (7)
C7—C2—C1—O2	−6.2 (6)	C45—C44—C43—F5	−179.7 (4)
C1—C2—C3—F1	−7.9 (7)	C45—C44—C43—C42	−0.9 (7)
C1—C2—C3—C4	174.4 (5)	C47—C44—C43—F5	0.5 (7)
C7—C2—C3—F1	174.6 (4)	C47—C44—C43—C42	179.2 (4)
C7—C2—C3—C4	−3.2 (7)	C43—C44—C47—O11	−176.7 (5)
C1—C2—C7—C6	−175.2 (4)	C43—C44—C47—O12	3.8 (7)
C3—C2—C7—C6	2.5 (7)	C45—C44—C47—O11	3.5 (7)
F1—C3—C4—C5	−176.5 (4)	C45—C44—C47—O12	−176.0 (4)
C2—C3—C4—C5	1.3 (8)	C46—C45—C44—C43	0.5 (7)
C6—C5—C4—C3	1.4 (8)	C46—C45—C44—C47	−179.7 (5)
C7—C6—C5—C4	−2.0 (8)	C44—C45—C46—C41	0.2 (8)

### Hydrogen-bond geometry (Å, °)

**Table:**

*D*—H···*A*	*D*—H	H···*A*	*D*···*A*	*D*—H···*A*
N2—H2A···O1^i^	0.82 (5)	2.39 (5)	3.056 (6)	140 (5)
N2—H2B···O10^ii^	0.84 (6)	2.03 (5)	2.863 (6)	176.4 (5)
N4—H4A···O4^iii^	0.81 (5)	2.11 (5)	2.825 (5)	149 (5)
N4—H4B···O9^iv^	0.92 (7)	1.96 (7)	2.874 (6)	176 (7)
O12—H121···O11^v^	0.92 (8)	1.71 (8)	2.626 (5)	174 (6)

Symmetry codes: (i) −*x*, *y*+1/2, −*z*+1/2; (ii) *x*, *y*+1, *z*; (iii) −*x*+1, *y*−1/2, −*z*+1/2; (iv) *x*, *y*−1, *z*; (v) −*x*+1, −*y*+2, −*z*.
